# The paradox of erectile dysfunction data after radical prostatectomy

**DOI:** 10.1590/S1677-5538.IBJU.2022.0289

**Published:** 2022-07-04

**Authors:** Tomás Bernardo Costa Moretti, Leonardo Oliveira Reis

**Affiliations:** 1 Universidade Estadual de Campinas – Unicamp Campinas SP Brasil UroScience, Universidade Estadual de Campinas – Unicamp, Campinas, SP, Brasil; 2 Pontifícia Universidade Católica de Campinas - PUC Campinas Campinas SP Brasil Pontifícia Universidade Católica de Campinas - PUC Campinas, Campinas, SP, Brasil

*To the editor,*

Who have never been approached in peer-reviewed journals or conference panel discussions on post-prostatectomy erectile dysfunction (PPED) with following questions: “Which assessment to apply? Has it been validated for a specific language? Self-applied or performed by another professional? Does it involve quality of life assessment and partner satisfaction?”.

These heterogeneities might limit more accurate comparisons of PPED rates among the three main techniques: open (ORP), laparoscopic (LRP) and robot-assisted radical prostatectomies (RARP) (1). Knowing the existence of a multifactorial influence on PPED and the great discrepancy among the studies, systematic reviews (SR) on this subject are still criticized for not being able to eliminate such allocation biases (2).

Although 14 types of review studies are described, all are monitored by a concept of sample homogeneity and evidence hyperfiltration to avoid spurious comparisons. However, the era of large databases and infinite amount of information has brought the need to analyze heterogeneous population data from the real world (3).

From this concept, a methodology developed by our study group, called Reverse Systematic Review (RSR) was born. In short, we started with the greatest evidence on a subject, the SR, and collected all the data found in the primary studies in order to generate a heterogeneous enough population-based database to bring together different scenarios where a surgical technique evidence was developed; in this case, radical prostatectomy (RP) (3, 4).

Thus, we applied RSR to understand how the main ED criteria were used throughout the natural history of RP techniques in order to allow a critical analysis of the literature. Through a systematic search carried out in December 2020, in 8 databases (PubMed, Web of Science, Cochrane Library, Embase, ProQuest, CINAHL, BVS/Bireme and Scopus), we selected 80 SR studies on radical prostatectomy (ORP, LRP and RARP) in a period between 2000/01/01 and 2020/12/05. When analyzing all the primary studies used in these SR, we found a total of 406 cohorts (nc= number of cohorts) that evaluated PPED using two most cited criteria: “Erection Sufficient for Intercourse” (ESI) and “Sexual Health Inventory for Men” (SHIM).

Among 406 cohorts corresponding to 118,994 patients (np= number of patients), 305 (75.1%) used the ESI and 101 (24.9%) used the SHIM. Among the group that used SHIM score, we subdivided it into categories regarding the degree of erectile dysfunction: moderate ED [SHIM 8-11] (nc=4; 1.0%); mild to moderate ED [SHIM 12-16] (nc=28; 6.9%); mild ED [SHIM 17-21] (nc=59; 14.5%) and no ED [SHIM 22-25] (nc=10; 2.5%).

The overall rate of sexual potency regardless of the criterion used was 25.5% (np=14,238; SE= 0.12) at 1 month, 33.6% (np =33,416; SE=0.09) at 3 months, 46.6% (np=41,936; SE= 0.09) at 6 months and 53.3% (np =78,089; SE=0.07) at 12 months.

**Figure 1:**
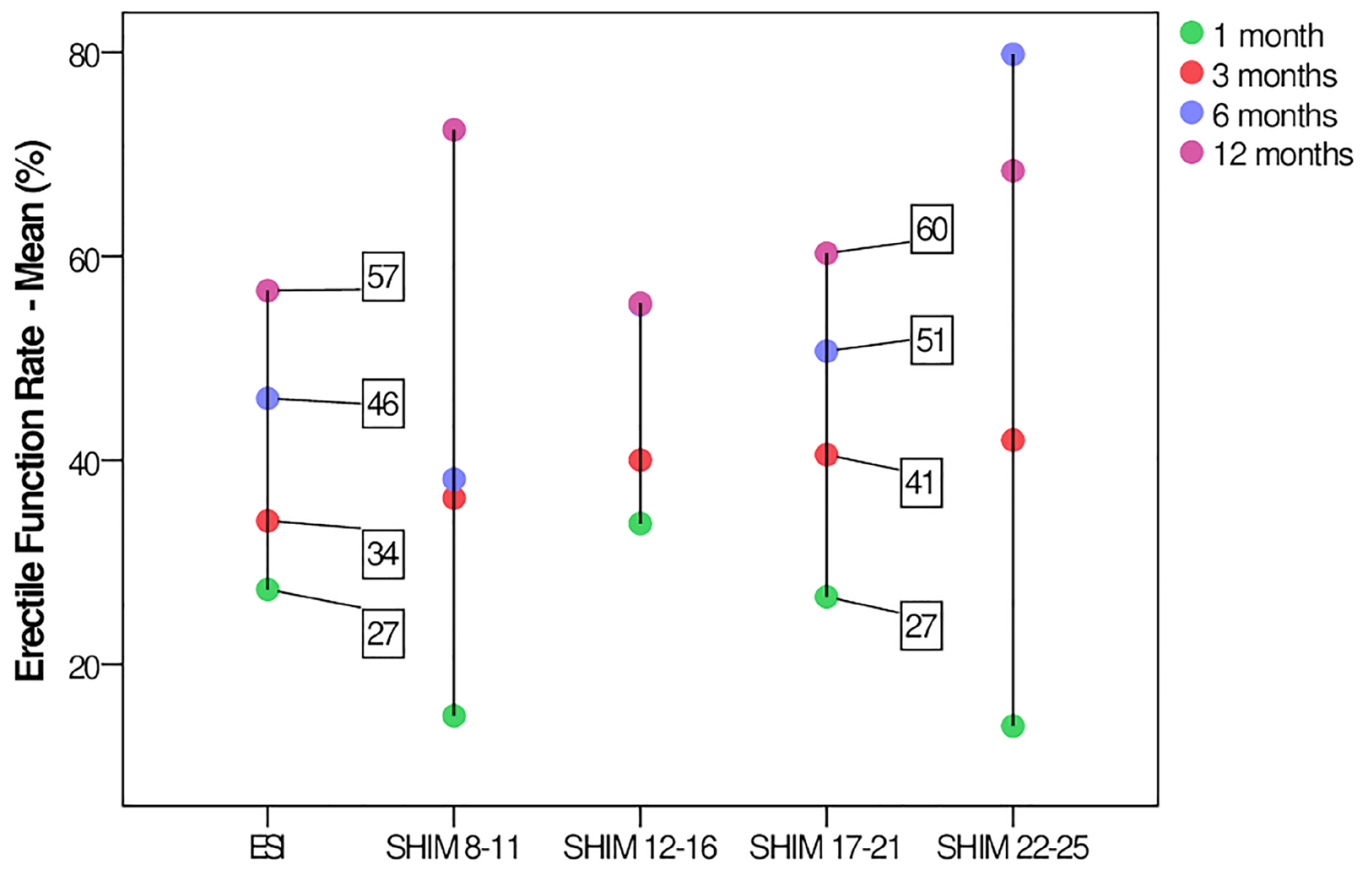
Erectile function recovery rates over time (colored dots) stratified by different definition criteria

A graphical representation of the mean values found in these studies was performed for each analysis period after surgery (1, 3, 6 and 12 months) according to the classification criteria listed above (Figure-1). In the graph, it is noted that the two lines that present a proportional distribution of the points are from the ESI and the SHIM 17-21, demonstrating that the results of these two assessments are closer and corresponding. The two most commonly used criteria reflect the same assessment intent to measure an “acceptable” degree of PPED.

Despite the ESI criterion having presented worse results when compared to the other criteria, it was the most used by the authors in 20 years of analysis. This demonstrates how the difficulty of application, validation and reproducibility of scores at an international level can influence scientists’ choices throughout RP natural history. Obviously, a field is not just influenced by science, but by the lack of it, and the acceptance of less rigorous and non-standard criteria by leading researchers might determine the available evidence, which creates a precedent for the scientific community, endorsing the use of a much-criticized evaluation criteria of sexual function.

This is the capacity that scientific influencers as a small group of prolific researchers on the issue have in the rest of the scientific community, which uses Cartesian arguments to criticize the works on erectile dysfunction and the lack of standardization of studies, but in practice applies the most comfortable and simple ED criterion. Interestingly, according to our methodology, different criteria might overlap.

After 20 years of coexistence between the three radical prostatectomy techniques (ORP, LRP and RARP) and much discussion, including among others (5) the best ED criteria to use, one sentence can summarize the state of art: “In practice, the theory is different”.

The Author
